# The Role of PET Imaging in the Differential Diagnosis between Radiation Necrosis and Recurrent Disease in Irradiated Adult-Type Diffuse Gliomas: A Systematic Review

**DOI:** 10.3390/cancers15020364

**Published:** 2023-01-05

**Authors:** Gaia Ninatti, Cristiano Pini, Fabrizia Gelardi, Martina Sollini, Arturo Chiti

**Affiliations:** 1 Residency Program in Nuclear Medicine, School of Medicine and Surgery, University of Milano Bicocca, 20900 Monza, Italy; 2 Department of Biomedical Sciences, Humanitas University, Via R. Levi Montalcini 4, 20090 Pieve Emanuele, Italy; 3 Humanitas Research Hospital, Department of Nuclear Medicine, Via Manzoni 56, 20089 Rozzano, Italy

**Keywords:** adult-type diffuse gliomas, primary brain tumours, PET

## Abstract

**Simple Summary:**

Adult-type diffuse gliomas are the most common primary brain tumours. Radiotherapy is one of the therapeutic options offered upfront and/or after surgery both in naïve and recurrent patients. Magnetic resonance imaging (MRI) is the recommended technique for treatment response evaluation and follow-up. However, in patients previously treated with radiotherapy, MRI might be difficult to interpret since recurrent disease and treatment-related changes often appear similar. Therefore, other imaging modalities, including positron emission tomography (PET), have been explored in this clinical setting. The present work focused on PET imaging in adult-type diffuse gliomas aims to review available literature data and assess the capability of PET in discriminating between radiation necrosis and disease relapse in irradiated patients.

**Abstract:**

Adult-type diffuse gliomas are treated with a multimodality treatment approach that includes radiotherapy both in the primary setting, and in the case of progressive or recurrent disease. Radiation necrosis represents a major complication of radiotherapy. Recurrent disease and treatment-related changes are often indistinguishable using conventional imaging methods. The present systematic review aims at assessing the diagnostic role of PET imaging using different radiopharmaceuticals in differentiating radiation necrosis and disease relapse in irradiated adult-type diffuse gliomas. We conducted a comprehensive literature search using the PubMed/MEDLINE and EMBASE databases for original research studies of interest. In total, 436 articles were assessed for eligibility. Ten original papers, published between 2014 and 2022, were selected. Four articles focused on [^18^F]FDG, seven on amino acid tracers ([^18^F]FET *n* = 3 and [^11^C]MET *n* = 4), one on [^11^C]CHO, and one on [^68^Ga]Ga-PSMA. Visual assessment, semi-quantitative methods, and radiomics were applied for image analysis. Furthermore, 2/10 papers were comparative studies investigating different radiopharmaceuticals. The present review, the first one on the topic in light of the new 2021 CNS WHO classification, highlighted the usefulness of PET imaging in distinguishing radiation necrosis and tumour recurrence, but revealed high heterogeneity among studies.

## 1. Introduction

Adult-type diffuse gliomas are the most common primary central nervous system (CNS) tumours and typically occur in adults. The most recent 2021 CNS WHO classification divides them into three types according to histological and molecular features, i.e., IDH mutant astrocytoma, IDH-mutant and 1p/19q-codeleted oligodendroglioma, and IDH-wildtype glioblastoma [1]. Patients with adult-type diffuse gliomas require a multimodal treatment approach usually combining surgery ± radiotherapy and/or systemic therapy [2]. Radiotherapy finds its application both in the primary setting, as adjuvant therapy or as first-line treatment in non-operable tumours, and in the case of progressive or recurrent disease. Radiation necrosis is a major complication of radiotherapy that might occur from some months to several years after the treatment completion. This radiation effect follows a combination of white matter damage and vascular injury, which results in increased capillary permeability and oedema, and vessel hyalinization with consequent thrombosis, ischemia, and tissue necrosis [3,4]. The rate of radiation necrosis ranges from 5% to 25% depending on the irradiated tumour volume, the dose, the fractionation, and any concurrent or sequential pharmacological therapy [5]. It is often irreversible and progressive, in contrast to early and mainly transient post-radiation changes such as pseudo progression [6]. Radiation necrosis may appear as a new gadolinium-enhancing lesion or signal abnormality on follow-up brain MRI, and thus mimic tumour recurrence [6,7]. Recurrent disease and treatment-related changes are indeed often indistinguishable using conventional imaging methods, and surgical sampling or close follow-up are needed to obtain a definitive diagnosis, which is of paramount importance to inform the appropriate patient management while avoiding diagnostic delays or unnecessary treatments. Growing evidence shows that advanced neuroimaging modalities may help to solve this diagnostic challenge; in particular, positron emission tomography (PET) with different radiopharmaceuticals has emerged as an useful modality to distinguish radiation necrosis and tumour recurrence, outperforming contrast enhanced MRI in most scenarios [8,9,10]. Many PET radiopharmaceuticals including among others those evaluating glucose consumption and cell membrane metabolism, amino acid transport, DNA synthesis, and neovasculature have been studied for this purpose [11,12]. Moreover, the recent advent of advanced image analysis techniques, such as radiomics and artificial intelligence, has the potential to further increase the sensitivity and specificity of PET imaging [13].

The present systematic review aims at defining the diagnostic accuracy of PET imaging using different radiopharmaceuticals and image analysis methods in discriminating radiation necrosis and disease relapse in irradiated adult-type diffuse gliomas as defined by the new 2021 CNS WHO classification.

## 2. Materials and Methods

Our review was registered in the international prospective register of systematic reviews PROSPERO (ID: CRD42022334795) and was carried out following the Preferred Reporting Items for Systematic Reviews and Meta-Analyses (PRISMA) [14] guidelines.

### 2.1. Search Strategy

We conducted a comprehensive literature search using the PubMed/MEDLINE and EMBASE databases for original research studies of interest. The following combination of terms was used: (“PET” OR “positron emission tomography”) AND (“radiation necrosis” OR “radionecrosis”) AND (“glioma” OR “brain” OR “glial” OR “astrocytoma” OR “oligodendroglioma” OR “glioblastoma”). The search was extended until 15 December 2022.

### 2.2. Eligibility Criteria and Study Selection

Two authors (GN and CP) independently performed a preliminary screening of retrieved titles and abstracts. After the removal of duplicates, we used the following exclusion criteria: (a) review articles and meta-analyses, guidelines, letters or commentaries, editorials, conference proceedings, and book chapters; (b) case reports and studies with <20 subjects; (c) studies not involving humans; (d) papers outside the field of interest; (e) articles without an available English translation. Any disagreement in the initial screening process was solved by a third reviewer (MS) using the majority vote. We then screened the full-text of selected papers using the following exclusion criteria: (f) studies involving patients with adult-type diffuse gliomas and other primary or metastatic brain tumours without available separate analyses; (g) suspected salami publication/slicing. Finally, other potentially relevant studies were searched among references of the retrieved full-text articles.

### 2.3. Data Extraction and Analysis

A database was created for the synthesis of included papers where the following information was collected: number of patients and lesions, radiopharmaceutical(s), glioma type distribution, image analysis method(s), neuropathological confirmation, time interval between radiation therapy and PET scan, and main results with metrics. In cases where more than one radiopharmaceutical was studied, results were reported and discussed separately. When multiple image analysis techniques were used, metrics were detailed for each method. The synthesis and analysis of included studies was done with Excel 2017 (Microsoft, Redmond, WA, USA).

### 2.4. Analysis of Quality

The QUADAS-2 tool [15] was used to determine the risk of bias and the applicability to the research question of each selected study. Two reviewers (GN and MS) independently analysed the quality of selected papers. Any disagreement was solved by a third reviewer (CP) using the majority vote.

## 3. Results

### 3.1. Study Selection

**Figure 1:**
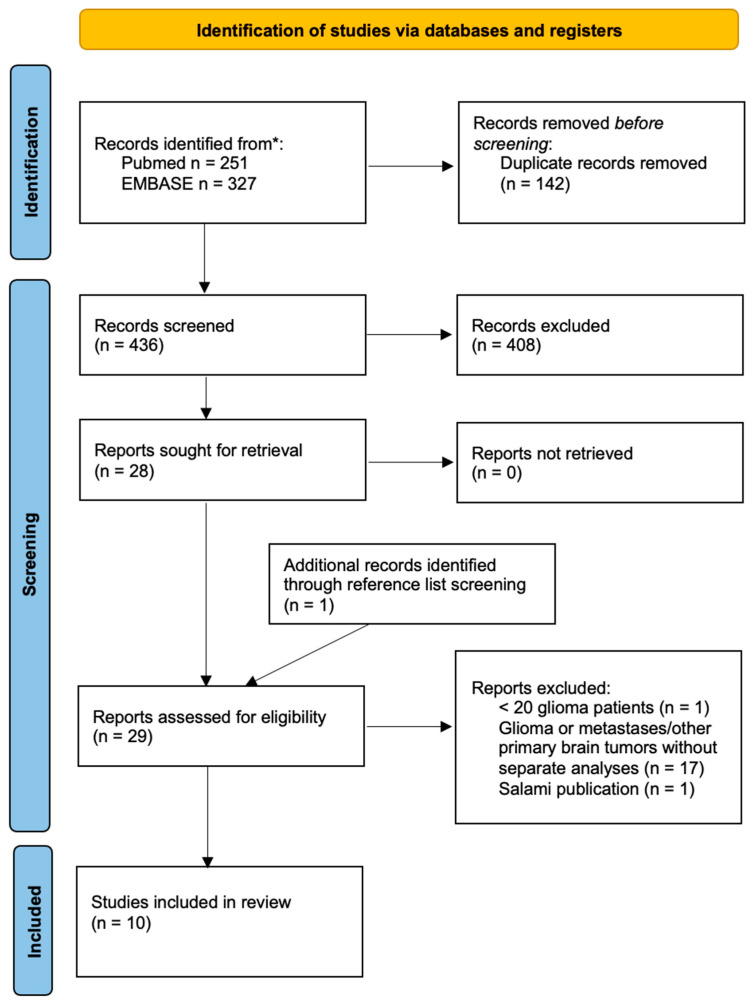
Summary of the article selection process. * Search performed up to 15 December 2022.

The search in the PubMed/MEDLINE and EMBASE databases returned 251 and 327 papers, respectively. After the removal of 142 duplicate records, 436 articles were assessed for eligibility by screening of titles and abstracts first, and of full texts in a later phase. One additional article was identified by screening reference lists of retrieved records. According to the above-mentioned criteria, a total of ten records were finally selected and included in the present review. A flow diagram summarizing the article selection process is reported in Figure 1.

### 3.2. Study Characteristics and Risk of Bias within Studies

**Figure 2:**
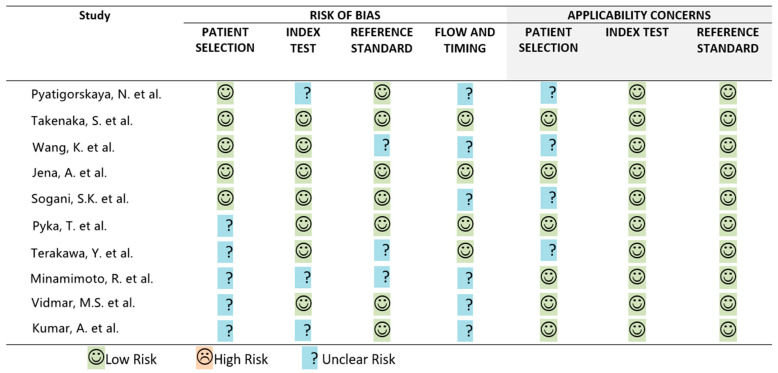
QUADAS.2 results.

Ten original research studies investigating the usefulness of brain PET in the differentiation of disease recurrence and radiation necrosis in 474 patients with irradiated adult-type diffuse gliomas, published between 2014 and 2022, were included in the review. The median number of lesions per study was 41 (range 32–63); five studies did not specify the number of lesions. Studies focused on different radiopharmaceuticals, including [^18^F]FDG (*n* = 4), amino acid tracers such as [^11^C]MET (*n* = 4) and [^18^F]FET (*n* = 3), [^11^C]CHO (*n* = 1), and [^68^Ga]Ga-PSMA (*n* = 1), and used a variety of image analysis methods, i.e., visual, semi-quantitative, and radiomics. Neuropathological confirmation was obtained in all patients in one study, partially in five, and was not mentioned in the remaining four. The time interval between radiation therapy and PET examination was specified only in three papers, and the overall median value was 35 months. The main characteristics of included studies are summarized in Table 1. The quality assessment of selected studies is summarized in Figure 2.

### 3.3. Main Results

#### 3.3.1. Imaging of Glucose Metabolism: [^18^F]FDG

Four studies [16,17,18,19] evaluating the ability of [^18^F]FDG to differentiate recurrence and radionecrosis reported accuracy values ranging from 63% to 88% (Table 2).

Heterogeneity in terms of image analysis methods, number of patients, and glioma type distribution most likely accounts for this variability in accuracy values. In the study by Pyatigorskaya et al. [16], semi-quantitative assessment using a TBRmax cutoff value of 2.44 outperformed visual analysis of [^18^F]FDG PET/CT images (accuracy of 77% versus 63%, respectively). Takenaka et al. [17] evaluated [^18^F]FDG PET/CT semi-quantitative parameters and showed that a TBRmax cutoff value of 1.26 was able to discriminate recurrent disease from radiation necrosis with good sensitivity and specificity (77% and 75%, respectively). Similar results were achieved using PET/MRI by Jena et al. [19] (88% for both a cutoff of 1.579 for TBRmax and of 1.179 for TBRmean). Wang et al. [18] showed excellent performance of an eight-textural features radiomic model in a large cohort of patients imaged by [^18^F]FDG PET/CT (AUC of 0.868 and 0.810 in the primary and validation cohorts, respectively).

#### 3.3.2. Amino Acid Tracers: [^18^F]FET and [^11^C]MET

The majority of included papers assessed the usefulness of radiolabelled amino acids for the differential diagnosis between tumour recurrence and radiation necrosis [17,18,20,21,22,23,24]. Of these, three studies focused on [^18^F]FET (Table 3a) and four on [^11^C]MET (Table 3b).

**Figure 3:**
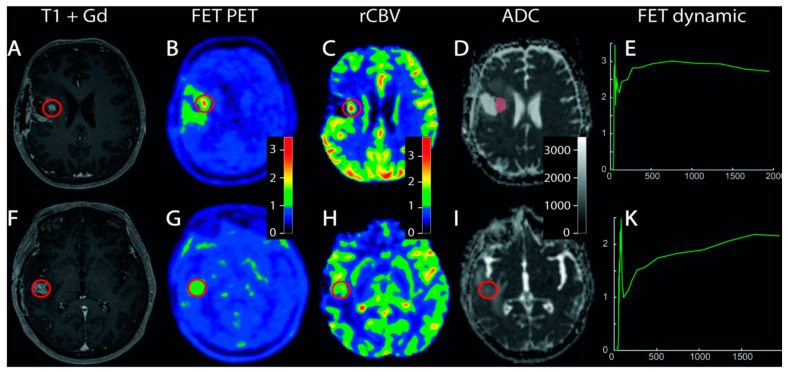
The increased uptake and early time to peak at [^18^F]FET PET/MRI allowed discriminating of tumour tissue in a patient with operated and irradiated anaplastic astrocytoma and a suspicious contrast-enhancing lesion appeared at MRI during follow-up (**A**–**E**). No significant uptake and late time to peak was observed in an area of contrast-enhancement at MRI in a patient with radio-chemo-treated glioblastoma, suggesting radiation necrosis (**F**–**K**). Reprinted/adapted with permission from Ref. [21]. Copyright © 2018 Elsevier B.V.

Collectively, there was a high variability in methods and metrics that prevented summarizing of the findings. Sogani et al. [20] prospectively tested static [^18^F]FET-derived semi-quantitative parameters and reported higher accuracy for the TBRmax cutoff value of 2.09 than for the TBRmean cutoff value of 1.517 (94% and 88%, respectively). The same was observed in the study by Vidmar et al. [24] even if they reported higher TBRmax and TBRmean cutoff values (3.03 and 2.04, respectively). The high discriminatory accuracy of TBRmax at [^18^F]FET PET/CT was confirmed by Pyka et al. [21] (Figure 3), who found that a TBRmax cutoff value of 2.07 30–40 min post-injection was successful for discriminating recurrent disease and radionecrosis (76% sensibility and 85% specificity).

Concerning [^11^C]MET, Takenaka et al. [17] showed that TBRmax with a cutoff of 2.51 had a 91% sensibility and 88% specificity. This finding was not confirmed by Terakawa et al. [22], who evidenced no statistically significant difference in TBRmax between recurrence and radionecrosis; the same authors observed that the only parameter with a predictive value was TBRmean (75% sensibility and 75% specificity using a cutoff of 1.58). Similarly, Minamimoto et al. [23] reported a low accuracy of [^11^C]MET TBRmax (AUC = 0.59 using a cutoff of 1.8); in the same study, visual analysis led to better results (AUC = 0.65). Finally, Wang et al. [18] built a predictive model consisting of five [^11^C]MET PET-derived radiomic features with an AUC of 0.767 and 0.750 in the primary and validation cohort, respectively.

#### 3.3.3. Targeting Cell Membrane Metabolism: [^11^C]CHO

Only one paper focused on [^11^C]CHO PET/CT (Table 4). The group found that a TBRmax cutoff of 8.92 was able to differentiate recurrence and radionecrosis with a sensitivity of 74% and a specificity of 88% [17].

#### 3.3.4. Targeting Glutamate Carboxypeptidase II (Prostate-Specific Membrane Antigen): [^68^Ga]Ga-PSMA-11

Kumar et al. [25] prospectively investigated the role of [^68^Ga]Ga-PSMA-11 PET/CT in the detection of recurrent tumours (Table 5). Recurrent patients exhibited high radiopharmaceutical uptake (median TBRmax 36.1, IQR 22.2–55.3), differently from patients with radiation necrosis (median TBRmax 1.08).

## 4. Discussion

The present review confirmed the usefulness of PET in distinguishing radiation necrosis and tumour recurrence in irradiated adult-type diffuse gliomas as defined by the new 2021 CNS WHO classification. The differentiation between radiation necrosis and recurrence in irradiated gliomas is a relatively common diagnostic conundrum, considering the prevalence of the disease and the key role radiotherapy plays in its treatment. The challenges this task poses often generate undecidedness on the subsequent patient management and may demand additional invasive investigations or a longitudinal follow-up that can hinder timely treatment. The relevance of the issue reverberates in an on-going florid scientific activity and literary production on the topic. Yet it may be questioned why if evidence about the potential role of PET arose since the 1980s [26,27], a definite answer about the role of PET is still lacking and a full consensus on the best tracer option is still debated. Indeed, relatively small cohorts of heterogenous patients often comprising many glioma types, retrospective design, different methods, and radiopharmaceuticals, ended up in a high fragmentation, which prevents meta-analysis of data and clearly demonstrates the benefit of nuclear medicine imaging in this clinical setting. Moreover, comparative studies assessing more than one radiopharmaceutical are few.

Most of the studies included in our review [16,17,18,19] evaluated [^18^F]FDG PET through semi-quantitative parameters reaching a high level of accuracy (77–88%) and converging to quite similar proposed cutoff values (TBR_max_ cutoff from 1.26 to 2.44). Although [^18^F]FDG exhibits a very high physiological brain uptake, it is widely available and radiation necrosis usually appears as hypometabolic. Furthermore, the advent of hybrid PET/MRI images [19] and advanced image analysis methods [18] renewed its role in brain tumour imaging. Nonetheless, [^18^F]FDG PET due to the high background and relatively low uptake of the tracer in some specific histological subtypes, especially by lower-grade gliomas [28], demonstrates some limitations in discriminating between radionecrosis and disease relapse. Delayed images are able to increase the target-to-background contrast, finally improving diagnostic performance [12].

The capability of amino acid tracers in distinguishing glioma recurrence from radiation necrosis has been confirmed by studies included in our analysis. Interestingly, two of the three studies focused on [^18^F]FET [20,21,24] identified comparable TBR_max_ cutoff values in this regard (2.07 [21] and 2.09 [20], respectively). On the other hand, the selected studies on [^11^C]MET PET showed favourable yet partially contrasting results in terms of accuracy of the method. Notably, the paper by Takenaka et al. [17] was the only paper among the included ones with a full pathological confirmation of the findings, and reported the best performance (AUC of 0.925) among the studies considered for the present review. The adoption of semi-quantitative tools is still debated, with discrepancies between advised cutoff values [17,22], while one study [23] showed a better performance of visual analysis alone. [^11^C]MET PET-derived radiomic features were able to differentiate glioma recurrence from radiation necrosis (AUC = 0.75 in the validation cohort) [18]. Although promising, these results should be interpreted with caution because of the heterogeneity of the cohort (43 GBM vs. 117 grade 2 and grade 3 nos gliomas) [18], and the well-known issues related to radiomic analysis in terms of methods, robustness, and reproducibility [29]. Specifically, none of the advanced imaging analyses (i.e., radiomics and artificial intelligence) models proposed and evaluated in brain tumours for differentiating pseudoprogression and radiation necrosis from true tumour progression have been prospectively validated [30]. Despite promising results, the use of [^11^C]MET is confined to referral centres by the necessity of an on-site cyclotron. The same production requirement is shared by [^11^C]CHO, evaluated in only one [17] of the selected studies. However, the sensitivity of [^11^C]CHO was lower than that reported for [^11^C]MET (74% [17] versus 75% [18,22], 81% [23], and 91% [17]). Notwithstanding differences in availability, type of analyses (visual or semi-quantitative), or in readers’ experiences with one or the other tracer, less evidence about the role of [^11^C]CHO compared to [^11^C]MET has been reported, tipping the scale in favour of the latter at least in a direct comparison. Nonetheless, [^18^F]CHO, thanks to its longer decay time and easier availability, may represent a valid substitute to [^11^C]CHO and an alternative to amino acid tracers in this specific clinical setting, even if available literature data [31,32] are not focused on the new classification of gliomas. Among amino acid tracers, [^18^F]FET has some advantages compared to [^11^C]MET including a longer half-life, the absence of radiolabelled metabolites, and a lower uptake in inflammation [33]. PSMA, a transmembrane protein overexpressed in different tumour types including gliomas [34], was recently investigated by Kumar et al. [25] in a compelling prospective study. [^68^Ga]Ga-PSMA-11 PET showed stunning accuracy with an excellent ability to discriminate between relapse and radionecrosis both visually and semi-quantitatively. This encouraging finding certainly deserves further thorough exploration, with particular regard to better evaluation of possible differences between GBM—representing almost two thirds of the considered study’s cohort—and other glioma types.

**Figure 4:**
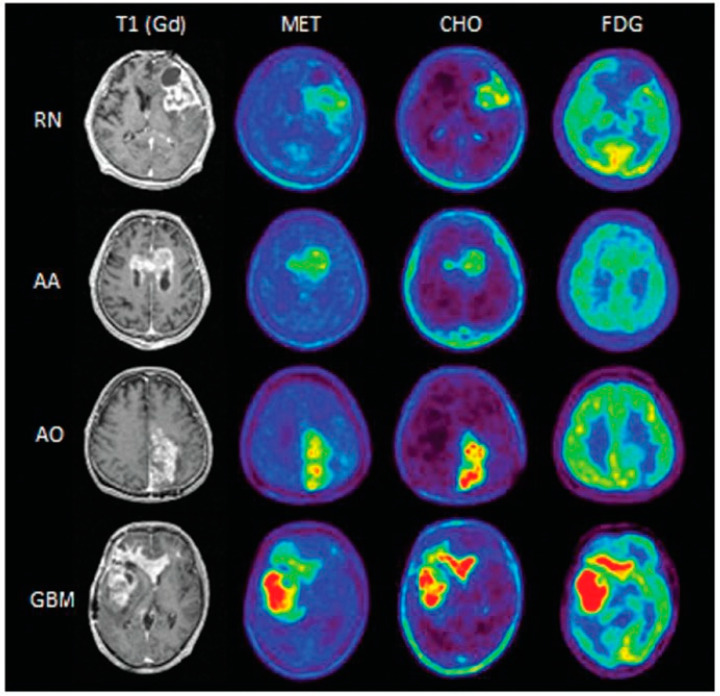
Comparison of the appearance of the findings of radiation necrosis (RN), anaplastic astrocytoma (AA), anaplastic oligodendroglioma (AO), and glioblastoma (GBM) between the different methods. Reprinted/adapted with permission from Ref. [17]. Copyright © 2014 The Japan Neurosurgical Society.

Only two of the ten papers (20%) included in the present review were comparative studies investigating different radiopharmaceuticals. Takenaka et al. [17] demonstrated the superiority of [^11^C]MET (AUC = 0.92) over [^11^C]CHO and [^18^F]FDG (AUC of 0.81 and 0.77, respectively), as depicted in Figure 4. Conversely, more recently Wang et al. [18] found that the radiomic signature extracted from [^18^F]FDG PET outperformed the one derived from [^11^C]MET images (AUC = 0.81 versus 0.75). However, the above-mentioned limitations of this study prevented further speculations regarding the best tracer and the role of advanced image analysis in this clinical setting.

To our knowledge, this is the first systematic review assessing the usefulness of PET in the differentiation between radiation necrosis and recurrence in light of the new 2021 CNS WHO classification. In particular, our work focused specifically on the adult-type diffuse gliomas family, which resulted in few eligible papers. Indeed, most of the studies that have been published on the topic included in their analyses other glial tumours, such as pilocytic astrocytoma and ependymomas [35,36], now clearly considered to be part of different families by the new classification. Nonetheless, meta-analyses summarizing these heterogeneous works (Table 6) are generally coherent with our outcomes and the most solid evidence has been generated in high-grade gliomas. In the majority of the cases, amino acid tracers outperformed [^18^F]FDG in terms of both sensitivity (82–91% for [^18^F]FET and 78–93% for [^11^C]MET versus 70–84% for [^18^F]FDG) and specificity (78–95% for [^18^F]FET and 78–93% for [^11^C]MET versus 70–88% for [^18^F]FDG), while similar performance of [^18^F]FET and [^11^C]MET has been reported, with a slightly higher sensitivity of the former over the latter. Furthermore, some of these meta-analyses showed that other radiopharmaceuticals including [^18^F]FLT and [^18^F]DOPA have been successfully explored in this clinical setting (Table 6).

Considering the wide spectrum of primary brain tumours as a single entity may prevent to disclose the full potential of each radiopharmaceutical at our disposal and to understand which one should preferentially be used depending on the specific clinical question. For instance, in the case of adult-type diffuse gliomas, radiopharmaceuticals are characterized by distinct uptake patterns and thus have wide ranges of sensitivity and specificity according to the type under study [47,48,49,50], both at primary diagnosis and recurrence. Recognizing these differences by rejecting a one-size-fits-all approach may allow to reach more solid conclusions and provide tailored indications in every clinical context. Although the evidence in not sufficient to draw definitive conclusions, some suggestions regarding the performance of different radiopharmaceutical in different clinical settings can be postulated (Table 7).

Overall, as the present review documented, many radiopharmaceuticals show encouraging-to-excellent accuracy, with particularly favourable data for radiolabelled amino acids such as [^11^C]FET and [^11^C]MET, justifying their consolidated role in the clinical management of these patients. PSMA-based tracers, already in wide use in the theranostics of prostate cancer, may replicate their success in the adult-type diffuse glioma setting. Research is blooming, but sparse, and the scientific community will need to focus its efforts on studies with larger sample sizes, possibly prospective and multicentric, providing neuropathological confirmation of the results and including analyses stratified according to the 2021 WHO diffuse glioma type. 

## 5. Conclusions

The present review emphasised the informative power of PET imaging as an impactful tool to differentiate between radiation necrosis and disease recurrence in irradiated adult-type diffuse gliomas. The relevance of the issue reverberates in an on-going florid research activity on the topic; however, the lack of consensus regarding the best diagnostic approach reflects on an important heterogeneity in study designs, radiopharmaceuticals, and analytical methods employed. This fragmentation, as per the mythological serpent ouroboros, continues in itself as it tends to produce inharmonious studies not generating evidence robust enough to decidedly shift the clinical paradigms.

Accordingly, further inquiries satisfying these still unmet needs are required to unravel the ouroboros, to harness the impressive enlightening power of the diagnostic tools at our disposal displayed in the present review towards the shared goal of a direct, robust, and lasting contribution to the clinical management of glioma patients.

## Figures and Tables

**Table 1:** Summary of the characteristics of included studies.

Study Characteristics	Included Studies
	(*n* = 10)
Number of patients	
≥100	1
<100	9
Study type	
Prospective	2
Retrospective	8
Radiopharmaceuticals	
[^18^F]FDG	2
[^11^C]MET	2
[^18^F]FET	3
[^11^C]CHO	0
[^68^Ga]Ga-PSMA-11	1
Mixed	2
Image analysis	
Visual	0
Semi-quantitative	6
Radiomics	1
Mixed	3
Neuropathological confirmation	
Yes	1
No	0
Partial	5
Not specified	4

**Table 2:** Summary of studies investigating the diagnostic accuracy of [^18^F]FDG PET.

Study	N of Patients (*n* of Lesions)	Glioma Type Distribution	Image Analysis Method	Neuropathological Confirmation (%)	Main Results
[16]	22 (ns)	- 1 grade 3 IDH-mut astrocytoma - 4 grade 2 oligodendroglioma - 17 GBM	Visual Semi-quantitative	8/22 (36%)	- Visual: sens 61%, spec 75%, acc 63% - Semi-quantitative (TBR_max_ cutoff 2.44): sens 85%, spec 50%, acc 77%, AUC 0.690
[18]	160 (ns)	- 72 grade 2 nos - 45 grade 3 nos - 43 GBM	Radiomics	ns	- Primary cohort: sens 74%, spec 90%, acc 78%, AUC 0.868 - Validation cohort: sens 69%, spec 77%, acc 71%, AUC 0.810
[17]	50 (ns)	- 23 grade 3 IDH-mut astrocytoma - 12 grade 3 oligodendroglioma - 15 GBM	Semi-quantitative	50/50 (100%)	TBR_max_ cutoff 1.26: sens 77%, spec 75%, AUC 0.774
[19]	35 (41)	- 9 grade 3 IDH-mut astrocytoma - 7 grade 2 oligodendroglioma - 4 grade 3 oligodendroglioma - 15 GBM	Semi-quantitative	18/35 (51%)	- TBR_max_ cutoff 1.579: sens 93%, 72.7%, 87.8%, AUC 0.827 - TBR_mean_ cutoff 1.179: sens 90%, 81.8%, 87.8%, AUC 0.888

Acc: accuracy; AUC: area under the curve; GBM: glioblastoma; IDH-mut: IDH mutated; nos: not otherwise specified; ns: not specified; sens: sensitivity; spec: specificity; TBR_max_: maximum target/background ratio; TBRmean: mean target/background ratio.

**Table 3:** Summary of studies investigating the diagnostic accuracy of [^18^F]FET and [^11^C]MET PET.

Study	N of Patients (*n* of Lesions)	Glioma Type Distribution	Image Analysis Method	Neuropathological Confirmation (%)	Main Results
a—*[^18^F]FET*					
[20]	32 (32)	Ns	Semi-quantitative	12/32 (38%)	- TBR_max_ cutoff 2.09: sens 100%, spec 72%, acc 94%, AUC 0.886 - TBR_mean_ cutoff 1.517: sens 89%, spec 86%, acc 88%, AUC 0.886
[21]	46 (63)	- 2 grade 2 IDH-mut astrocytoma - 13 grade 3 IDH-mut astrocytoma - 1 grade 2 oligodendroglioma - 3 grade 3 oligodendroglioma - 27 GBM	Semi-quantitative	23/63 (37%)	- TBR_max_ at 10–20 min cutoff 1.71: sens 76%, spec 85%, AUC 0.848 - TBR_max_ at 30–40 min cutoff 2.07: sens 80%, spec 85%, AUC 0.863 - TTP 20 min: sens 64%, spec 79%, AUC 0.728
[24]	42 (ns)	Ns	Semi-quantitative	11/47 (23%)	- TBR_max_ cutoff 3.03: sens 77%, spec 82%, acc 79% - TBR_mean_ cutoff 2.04: sens 71%, spec 91%, acc 76%
b—*[^11^C]MET*					
[18]	160 (ns)	- 72 grade 2 nos - 45 grade 3 nos - 43 GBM	Radiomics	Ns	- Primary cohort: sens 73%, spec 69%, acc 72%, AUC 0.767 - Validation cohort: sens 75%, spec 69%, acc 74%, AUC 0.750
[17]	50 (ns)	- 23 grade 3 IDH-mut astrocytoma - 12 grade 3 oligodendroglioma - 15 GBM	Semi-quantitative	50/50 (100%)	TBR_max_ cutoff 2.51: sens 91%, spec 88%, AUC 0.925
[22]	26 (32)	- 6 grade 2 nos - 6 grade 3 nos - 14 GBM	Semi-quantitative	Ns	TBR_mean_ cutoff 1.58: sens 75%, spec 75%
[23]	31 (ns)	- 12 grade 3 IDH-mut astrocytoma - 19 GBM	Visual Semi-quantitative	Ns	- Visual: sens 81%, spec 50%, acc 71%, AUC 0.65 - Semi-quantitative (TBR_max_ cutoff 1.8): AUC 0.59

Acc: accuracy; AUC: area under the curve; GBM: glioblastoma; IDH-mut: IDH mutated; nos: not otherwise specified; ns: not specified; sens: sensitivity; spec: specificity; TBR_max_: maximum target/background ratio; TBRmean: mean target/background ratio.

**Table 4:** Summary of studies investigating the diagnostic accuracy of [^11^C]CHO PET.

Study	N of Patients (*n* of Lesions)	Glioma Type Distribution	Image Analysis Method	Neuropathological Confirmation (%)	Main Results
[17]	50 (ns)	- 23 grade 3 IDH-mut astrocytoma - 12 grade 3 oligodendroglioma - 15 GBM	Semi-quantitative	50/50 (100%)	TBR_max_ cutoff 8.92: sens 74%, spec 88%, AUC 0.814

Acc: accuracy; AUC: area under the curve; GBM: glioblastoma; IDH-mut: IDH mutated; ns: not specified; sens: sensitivity; spec: specificity; TBRmax: maximum target/background ratio.

**Table 5:** Summary of studies investigating the diagnostic accuracy of [^68^Ga]PSMA-11 PET.

Study	N of Patients (*n* of Lesions)	Glioma Type Distribution	Image Analysis Method	Neuropathological Confirmation (%)	Main Results
[25]	30 (49)	- 3 grade 3 oligodendroglioma - 8 grade 3 IDH-mut astrocytoma - 19 GBM	Visual Semi-quantitative	Ns	PET positive in all recurrent tumours, no significant radiopharmaceutical accumulation in patients with radiation necrosis—median TBR_max_ recurrent tumours 36.1 (IQR 22.2–55.3) vs. radiation necrosis 1.08

Acc: accuracy; GBM: glioblastoma; IDH-mut: IDH mutated; IQR: interquartile range; ns: not specified; sens: sensitivity; spec: specificity; TBRmax: maximum target/background ratio; TBRmean: mean target/background ratio; vs: versus.

**Table 6:** Summary of the main results of meta-analyses evaluating the role of PET in the differential diagnosis between radiation necrosis and disease recurrence in glial tumours.

Reference	[^18^F]FDG	[^11^C]MET	[^18^F]FET	[^11^C]Choline	[^18^F]DOPA	[^18^F]FLT
Nihashi et al., 2013 [37]	Sens 77% (95% CI: 66–85%), spec 78% (95% CI: 54–91%)	* Sens 70% (95% CI: 50–84%), spec 93% (95% CI: 44–100%)	Ne	Ne	Ne	Ne
Deng et al., 2013 [38]	Ne	Sens 87% (95% CI: 81–92%), spec 81% (95% CI: 72–80%), AUC 0.8938	Ne	Ne	Ne	Ne
Wang et al., 2015 [39]	Sens 70% (95% CI: 64–75%), spec 88% (95% CI: 80–93%), AUC 0.8661	Sens 85% (95% CI: 76–91%), spec 83% (95% CI: 71–92%), AUC 0.8914	Ne	Ne	Ne	Ne
Li et al., 2015 [40]	Sens 78% (95% CI: 69–85%), spec 77% (95% CI: 66–85%), AUC 0.84	Ne	Ne	Ne	Ne	Sens 82% (95% CI: 51–95%), spec 76% (95% CI: 50–91%), AUC 0.85
Xu et al., 2017 [41]	Ne	Sens 88% (95% CI: 85–91%), spec 85% (95% CI: 80–89%), AUC 0.9352	Ne	Ne	Ne	Ne
Yu et al., 2018 [42]	Ne	Ne	Sens 82% (95% CI: 79–84%), spec 80% (95% CI: 76–83%), AUC 0.8976	Ne	Sens 85% (95% CI: 81–88%), spec 77% (95% CI: 74–81%), AUC 0.8771	Ne
Gao et al., 2018 [43]	Ne	Ne	Ne	Sens 87% (95% CI: 78–93%), spec 82% (95% CI: 69–91%)	Ne	Ne
Furuse et al., 2019 [44]	Sens 79% (95% CI: 60–90%), spec 70% (95% CI: 58–81%)	Sens 79% (95% CI: 65–88%), spec 82% (95% CI: 68–91%)	Sens 91% (95% CI: 79–97%), spec 95% (95% CI: 61–99%)	Ne	Ne	Ne
De Zwart et al., 2020 [45]	* Sens 84% (95% CI: 72–92%), spec 84% (95% CI: 69–93%)	* Sens 93% (95% CI: 80–98%), spec 82% (95% CI: 68–91%)	* Sens 90% (95% CI: 81–95%), spec 85% (95% CI: 71–93%)	Ne	Ne	Ne
Cui et al., 2021 [46]	Sens 78% (95% CI: 71–83%), spec 87% (95% CI: 80–92%)	Sens 92% (95% CI: 83–96%), spec 78% (95% CI: 69–86%)	Sens 88% (95% CI: 80–93%), spec 78% (95% CI: 69–85%)	Ne	Sens 85% (95% CI: 80–89%), spec 70% (95% CI: 60–79%)	Ne

* Pooled data calculated in high-grade gliomas. AUC: area under the curve; CI: confidence interval; ne: not evaluated; sens: pooled sensitivity; spec: pooled specificity.

**Table 7:** Summary of the performance of different PET tracers and analyses according to glioma type.

Study	Glioma Type	N of Patients	Visual Analysis	Semi-Quantitative Analysis	Radiomic Analysis
[^18^F]FDG					
[16,17,18,19]	Grade 3 IDH-mut astrocytoma	∑ 33	++	++	+/−
	Grade 2 oligodendroglioma	∑ 11	+	+	?
	Grade 3 oligodendroglioma	∑ 16	+	+	?
	GBM	∑ 90	++	++	+/−
[^18^F]FET					
[21]	Grade 2 IDH-mut astrocytoma	2	?	+/−	?
	Grade 3 IDH-mut astrocytoma	13	?	+	?
	Grade 2 oligodendroglioma	1	?	+/−	?
	Grade 3 oligodendroglioma	3	?	+/−	?
	GBM	27	?	++	?
[^11^C]MET					
[17,18,22,23]	Grade 3 IDH-mut astrocytoma	∑ 35	++	++	?
	Grade 3 oligodendroglioma	12	?	+	?
	GBM	∑ 91	++	++	+/−
[^11^C]CHO					
[17]	Grade 3 IDH-mut astrocytoma	23	?	+	?
	Grade 3 oligodendroglioma	12	?	+	?
	GBM	15	?	+	?
[^68^Ga]Ga-PSMA-11					
[25]	Grade 3 oligodendroglioma	3	+/−	+/−	?
	Grade 3 IDH-mut astrocytoma	8	+/−	+/−	?
	GBM	19	+	+	?

Based on literature data (number of patients, studies, and results), the application of each analysis for each tracer according to the glioma type was evaluated as ++: suitable; +: promising; +/−: undetermined; and ?: unknown.
